# Concurrent validity of provisional remission criteria for gout: a dual-energy CT study

**DOI:** 10.1186/s13075-019-1941-8

**Published:** 2019-06-21

**Authors:** Nicola Dalbeth, Christopher Frampton, Maple Fung, Scott Baumgartner, Savvas Nicolaou, Hyon K. Choi

**Affiliations:** 1 Bone and Joint Research Group, Department of Medicine, Faculty of Medical and Health Sciences, University of Auckland, 85 Park Rd, Grafton, Auckland, 1023 New Zealand; 2 University of Otago, Christchurch, New Zealand; 3 Formerly Ardea Biosciences, Inc., San Diego, CA USA; 4 Vancouver General Hospital and University of British Columbia, Vancouver, BC Canada; 5 Harvard Medical School and Massachusetts General Hospital, Boston, MA USA

**Keywords:** Gout, Urate, Outcome measures, Remission, Dual-energy CT

## Abstract

**Background:**

Provisional gout remission criteria including five domains (serum urate, tophus, flares, pain due to gout, and patient global assessment) have been proposed. The aim of this study was to test the concurrent validity of the provisional gout remission criteria by comparing the criteria with dual-energy CT (DECT) findings.

**Methods:**

Patients with gout on allopurinol ≥ 300 mg daily were prospectively recruited into a multicenter DECT study. Participants attended a standardized study visit which recorded gout flare frequency in the preceding 12 months, physical examination for tophus, serum urate, and patient questionnaires. DECT scans of both hands/wrists, feet/ankles/Achilles, and knees were analyzed by two DECT radiologists. The relationship between the DECT urate crystal volume and deposition with individual domains as well as the provisional remission criteria set was analyzed.

**Results:**

The provisional remission criteria were fulfilled in 23 (15.1%) participants. DECT urate crystal deposition was observed less frequently in those fulfilling the provisional remission criteria (44%), compared with those not fulfilling the criteria (73.6%, odds ratio 0.28, *P* = 0.004). The median (range) DECT urate crystal volume was 0.00 (0.00–0.46) cm^3^ for those fulfilling the remission criteria, compared with 0.08 (0.00–19.53) cm^3^ for those not fulfilling the criteria (*P* = 0.002). In multivariate regression analysis, the serum urate and tophus domains were most strongly associated with DECT urate crystal deposition.

**Conclusions:**

In people with gout established on allopurinol, a state of remission as defined by the provisional remission criteria is associated with less DECT urate crystal deposition. While this study provides support for the validity of the provisional gout remission criteria, it also demonstrates that some crystal deposition may be present in people achieving these criteria.

**Electronic supplementary material:**

The online version of this article (10.1186/s13075-019-1941-8) contains supplementary material, which is available to authorized users.

## Background

Gout is a chronic disease of monosodium urate (MSU) crystal deposition, which typically presents as intermittent flares of severe inflammatory arthritis [1, 2]. Long-term urate-lowering therapy can lead to dissolution of MSU crystals, with resultant prevention of gout flares, regression of tophi, and improved patient-reported outcomes [3–6].

Disease remission is the goal of therapy for many chronic rheumatic diseases; this state has been defined as “the absence of signs and symptoms attributable to a disease, when the symptoms and signs can return in the future, with the understanding that the momentary absence of signs and symptoms, particularly in conditions characterized by intermittent symptoms, does not equate to remission” [7]. Provisional domains and definitions for gout remission criteria have been proposed using consensus methodology [8]. These criteria include the following OMERACT-endorsed chronic gout domains [9]: serum urate, tophus, flares, pain due to gout, and patient global assessment. These criteria have been used to determine remission cut points for a gout disease activity score in a longitudinal study of 446 patients [10]. However, to date, the concurrent validity of the provisional gout remission criteria has not been tested.

Dual-energy CT (DECT) is an advanced imaging technique that allows color coding and volumetric measurement of MSU crystal deposition [11]. We have recently reported the results of a large multicenter DECT study of people with gout on allopurinol ≥ 300 mg daily for at least 3 months [12] and observed that high DECT urate crystal volumes were positively associated with serum urate levels, number of gout flares, tophi, and patient global assessment of disease activity. Here, we describe, in the same cohort, an analysis to test the concurrent validity of the provisional remission criteria by examining the association of individual remission domains and the full remission criteria set with DECT urate crystal deposition.

## Methods

### Study population and relevant variables

The clinical study methods have been previously reported in full [12]. In brief, patients with gout on allopurinol ≥ 300 mg daily for at least 3 months were prospectively recruited into a multicenter DECT study, using monitored enrollment to include approximately 25% of patients with subcutaneous tophi and 50% with serum urate < 0.36 mmol/L (6.0 mg/dL). All participants fulfilled the 1977 American Rheumatism Association gout classification criteria [13] and attended a standardized study visit, which recorded gout flare frequency in the preceding 12 months, physical examination for tophus, serum urate, and patient questionnaires. Patient assessment of gout disease activity (0–10 numerical rating scale, 0, none, 10, extremely active) and pain due to gout (numerical rating scale: 0, no pain; 10, severe pain) were recorded.

DECT of both hands/wrists, feet/ankles/Achilles, and knees were performed using the second-generation Siemens 128-slice Definition Dual Source scanner. We utilized kernels with integrated beam hardening correction. Urate crystal volume was measured by two DECT radiologists who were blinded to all clinical data. The radiologists were both imaging specialists with sub-specialization in dual-energy analysis for numerous applications with certification in DECT. The radiologists measured volumes independent of each other, and any cases where some discrepancy did exist were shared for consensus reading. Previous analysis from the same investigators has shown inter- and intra-reader intraclass correlation coefficients for DECT urate volumes of 1.00 (95% CI, 1.00 to 1.00) and 1.00 (95% CI, 1.00 to 1.00), with corresponding bias estimates (SD) of 0.01 (0.00) cm^3^ and 0.01 (0.03) cm^3^ [14]. Gout software (syngo.via VB10 software package, Siemens, Forchheim, Germany) used characteristic differences in attenuation at these voltages to produce digital color-coded images that rendered urate green. Green-rendered areas were required to have a minimum diameter of 3 mm to be described as urate positive, to reduce false positive assessments due to artifact [15]. Urate crystal volume was calculated using a dedicated automated volume assessment software program (syngo.via VB10 software package). The evaluation limits for urate volume assessment were set at − 1 for upper HU and − 1000 for lower HU.

### Remission criteria and variables

The following individual remission domains were analyzed: serum urate (serum urate < 0.36 mmol/L), tophus (absence), flares (none in the preceding 12 months), pain (pain due to gout score < 2), and patient global assessment (patient global assessment score < 2). The remission domains and definitions fully aligned with the provisional remission criteria, with the exception that measures of the serum urate, pain, and patient global assessment were recorded at a single time point rather than twice over a 12-month period as stated in the provisional remission criteria [8].

### Statistical analysis

Demographics and clinical features were summarized using standard descriptive statistics including means, SD, median, range, number, and percent as appropriate. The number of individual domains fulfilled was assessed using cumulative percentage plots. The relationship between the DECT urate crystal deposition and volume with each individual domain as well as with the full remission criteria set (all 5 domains met) were analyzed using chi-square and Mann Whitney *U* tests respectively, and in regression models (logistic regression for the presence of DECT urate crystal deposits and univariate analysis of variances for the rank of DECT urate crystal volume).

## Results

### Study participant and remission domain description

Clinical characteristics and individual remission domain results for all 152 participants are shown in Table 1. Participants were predominantly middle-aged men, with a mean disease duration of 12 years. The serum urate remission domain was fulfilled in 77 (50.7%) participants, tophus domain in 104 (68.4%), flare domain in 70 (46.1%), pain domain in 104 (68.4%), and patient global assessment domain in 84 (55.2%). All 5 remission domains were fulfilled in 23 (15.1%) participants, and none in 8 (5.3%) participants.

**Table 1:** Characteristics of participants according to the provisional remission criteria

	All participants (*n* = 152)	Provisional remission criteria fulfilled (*n* = 23)	Provisional remission criteria not fulfilled (*n* = 129)
Age (years), mean (SD)	58 (11)	62 (11)	58 (11)
Male, *n* (%)	140 (92.1)	20 (97)	120 (93.0)
Race, *n* (%)			
White	98 (64.5)	16 (70)	82 (63.6)
Non-white	54 (35.5)	7 (30)	47 (36.3)
Duration of gout (years), median (range)	12 (1–25)	10 (1–35)	12 (1–45)
Allopurinol daily dose, *n* (%)			
300 mg	124 (84.6)	21 (91)	103 (79.8)
> 300 mg	28 (18.4)	2 (9)	26 (20.2)
Duration of allopurinol use (years), median (range)	2.9 (0.2–45)	3.3 (0.3–27)	2.8 (0.2–45)
Serum urate (< 0.36 mmol/L)	77 (50.7)	23 (100)	54 (41.9)
Tophus (absence)	104 (68.4)	23 (100)	81 (62.8)
Flares (none in the last 12 months)	70 (46.1)	23 (100)	47 (36.4)
Pain (due to gout, < 2)	104 (68.4)	23 (100)	81 (62.8)
Patient global assessment of gout activity (< 2)	84 (55.2)	23 (100)	61 (47.3)

### Relationships between individual remission domains

**Fig. 1:**
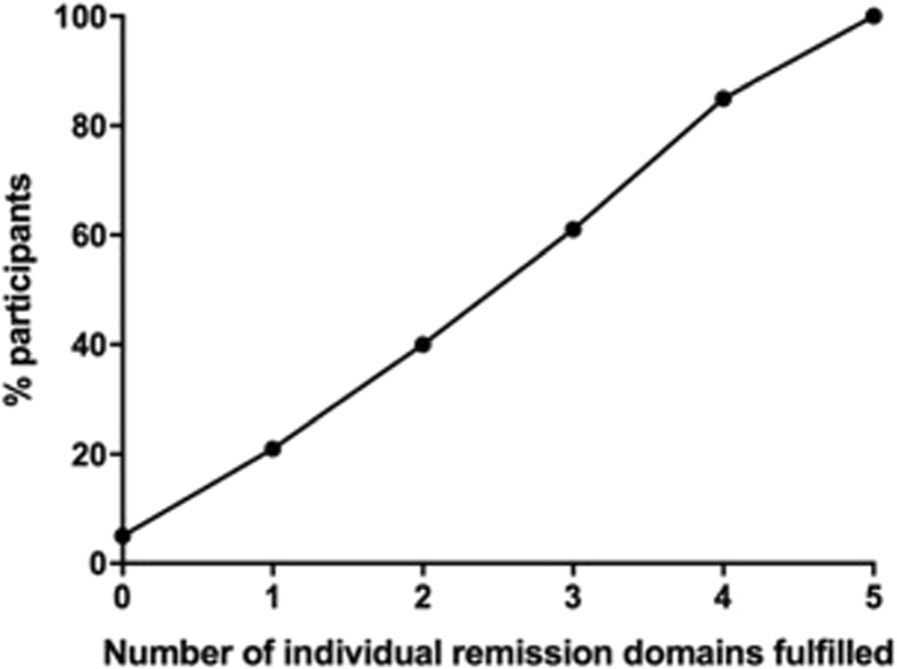
Cumulative percentage plot showing the distribution of participants fulfilling each number of individual remission domains

Some overlap was observed between the individual remission domains, with the highest overlap between the pain and patient global assessment domains (50.7%), and between the pain and tophus domains (48.0%), and the lowest overlap between the serum urate and flare domains (20.4%) (Additional file 1: Table S1 and S2). More than half of participants fulfilling the tophus remission domain (no tophi) did not fulfill any other individual remission domains, whereas < 10% of patients fulfilling the flare remission domain (none in the preceding 12 months) did not fulfill any other individual remission domains (Additional file 2: Figure S1). The cumulative percentage plot of the number of individual domains appeared linear, suggesting that all five individual domains contributed to the provisional remission criteria (Fig. 1).

### Presence of DECT urate crystal deposition and remission criteria

Fewer participants fulfilling the provisional remission criteria had DECT urate crystal deposition; DECT urate crystal deposition was present in 10/23 (44%) fulfilling the provisional remission criteria compared with 95/129 (73.6%) not fulfilling the provisional criteria (odds ratio 0.28, *P* = 0.004, Table 2). More than half of the participants fulfilling each individual domain criteria had DECT evidence of crystal deposition. For individual remission domains, fewer participants fulfilling the serum urate domain and the patient global assessment domain had DECT urate crystal deposition in univariate analysis (Table 2). In the logistic regression model which included all individual remission domains, DECT urate crystal deposition was independently associated with the serum urate domain (*P* = 0.001) and the tophus domain (*P* = 0.042, Table 3).

**Table 2:** Presence of DECT urate crystal deposition according to individual remission domains and the provisional gout remission criteria

Domain	*N* (%)with DECT urate crystal deposition for participants fulfilling individual domain/provisional criteria	*N* (%) with DECT urate crystal deposition for participants not fulfilling individual domain/provisional criteria	Odds ratio (95% CI)	*P*
Serum urate (< 0.36 mmol/L)	43 (56) *n* = 77	62 (83) *n* = 75	0.27 (0.13–0.56)	< 0.001
Tophus (absence)	67 (64) *n* = 104	38 (79) *n* = 48	0.48 (0.22–1.03)	0.07
Flares (none in the last 12 months)	44 (63) *n* = 70	61 (74) *n* = 82	0.58 (0.29–1.15)	0.13
Pain (due to gout, < 2)	70 (67) *n* = 104	35 (73) *n* = 48	0.76 (0.37–1.65)	0.49
Patient global assessment of gout activity (< 2)	52 (62) *n* = 84	53 (78) *n* = 68	0.46 (0.22–0.94)	0.03
Provisional remission criteria (all five domains)	10 (44) *n* = 23	95 (74) *n* = 129	0.28 (0.11–0.65)	0.004

**Table 3:** Relation between provisional remission domains and presence of DECT crystal deposition in multivariate regression model*

Variable	*B*	SE	Exp(*B*)	*P*
Serum urate (< 0.36 mmol/L)	− 1.40	0.41	0.25	0.001
Tophus (absence)	− 0.90	0.44	0.41	0.042
Flares (none in the last 12 months)	− 0.03	0.43	0.97	0.94
Pain (due to gout, < 2)	0.18	0.52	1.2	0.73
Patient global assessment of gout activity (< 2)	− 0.62	0.50	0.54	0.21

*All individual remission domains were forced into the model

### DECT urate crystal volume and remission criteria

The median (range) DECT urate crystal volume was 0.00 (0.00–0.46) cm^3^ for those fulfilling the provisional remission criteria, compared with 0.08 (0.00–19.53) cm^3^ for those not fulfilling the provisional remission criteria (*P* = 0.002, Table 4). With the exception of the pain domain, participants fulfilling each remission domain had lower DECT urate crystal volume than those who did not fulfill the domain in univariate analysis (Table 4). In the regression model of ranked DECT crystal volumes including all individual remission domains, DECT urate crystal volume was independently associated with the serum urate domain (*P* = 0.036), the tophus domain (*P* = 0.001), and the patient global assessment domain (*P* = 0.043, Table 5).

**Table 4:** DECT urate crystal volume according to individual remission domains and the provisional gout remission criteria

Domain	Median (range) of DECT urate crystal volume for participants fulfilling individual domain/provisional criteria	Median (range) of DECT urate crystal volume for participants not fulfilling individual domain/provisional criteria	*P*
Serum urate (< 0.36 mmol/L)	0.03 (0.00–4.63) *n* = 77	0.09 (0.00–19.53) *n* = 75	0.016
Tophus (absence)	0.05 (0.00–1.23) *n* = 104	0.21 (0.00–19.53) *n* = 48	0.001
Flares (none in the last 12 months)	0.05 (0.00–2.57) *n* = 70	0.11 (0.00–19.53) *n* = 82	0.011
Pain due to gout (< 2)	0.07 (0.00–5.11) *n* = 104	0.08 (0.00–19.53) *n* = 48	0.43
Patient global assessment of gout activity (< 2)	0.05 (0.00–3.34) *n* = 84	0.11 (0.00–19.53) *n* = 68	0.002
Provisional remission criteria (all five domains)	0.0 (0.00–0.46) *n* = 23	0.08 (0.00–19.53) *n* = 129	0.002

**Table 5:** Relation between provisional remission domains and DECT urate crystal volume in multivariate regression model*

Variable	Mean square	*F*	*P*
Serum urate (< 0.36 mmol/L)	7225	4.46	0.036
Tophus (absence)	18,257	11.27	0.001
Flares (none in the last 12 months)	2167	1.34	0.25
Pain (due to gout, < 2)	2100	1.30	0.26
Patient global assessment of gout activity (< 2)	6764	4.17	0.043

*All individual remission domains were forced into the model. DECT crystal volumes were analyzed as ranks. Model statistics: adjusted *R*^2^ = 0.14, *F* = 5.8, *P* < 0.001

### Serum urate cut points in the provisional remission criteria

There were 41 (27%) participants with serum urate < 5 mg/dL. Of these 41 participants, 14 (60.9%) fulfilled the provisional remission criteria and 27 (20.9%) did not fulfill the remission criteria. DECT urate crystal deposition was present in 23/41 (56%) participants with serum urate < 5 mg/dL, compared with 82/111 (73.9%) participants with serum urate ≥ 5 mg/dL (OR [95% CI] 0.45 [0.21–0.95], *P* < 0.001).

If the serum urate domain in the remission criteria was reduced to a level below 5 mg/dL, rather than 6 mg/dL, only 14 (9.2%) participants fulfilled the provisional remission criteria. Using the lower serum urate cut point, the relationship between the provisional remission criteria and DECT urate crystal deposition was similar; 6/14 (42.9%) participants fulfilling the provisional remission criteria had DECT urate crystal deposition, compared with 99/138 (71.7%) who did not fulfill the provisional remission criteria (OR [95% CI] 0.29 [0.10–0.91], *P* = 0.026).

## Discussion

This study has shown that in gout patients on urate-lowering therapy, those achieving a state of remission defined by the provisional remission criteria have less DECT urate crystal deposition. The individual remission domains most directly related to monosodium urate crystal deposition (serum urate and tophus) are independently associated with MSU crystal deposition measured by DECT. Furthermore, there was only a modest overlap between the different individual remission domains and all domains contributed to the remission criteria, supporting the selection of domains within the provisional remission criteria.

Prior research has shown that there is no uniformity in patient preferences for measurement of gout outcome domains and that different patient groups value different domains [16]. Consistent with these previous observations, the cumulative percentage analysis of individual domains in this study showed that each domain within the provisional criteria set contributed to the overall remission criteria. Overlaps between individual remission domains were variable, with the highest overlaps observed between the two patient-reported domains of pain and patient global assessment, and the pain and tophus domains. In contrast, the lowest overlap was observed between the serum urate and flare domains. The mismatch between serum urate and flares may represent the delay in time between achieving serum urate lowering and long-term suppression of gout flares. This finding emphasizes the additive benefit of measuring clinical outcomes as well as serum urate when assessing remission in gout.

It is noteworthy that while DECT measures of crystal deposition (both volume assessment and presence of deposition) were lower in those achieving a state of remission defined by the provisional remission criteria, 44% of participants achieving this state had some evidence of DECT urate crystal deposition. Furthermore, more than half of the participants fulfilling each individual domain criteria had DECT evidence of crystal deposition. These findings indicate that even in people with few clinical symptoms of disease, MSU crystal deposition can still be present. Recent studies have shown that urate crystal deposits identified by DECT are responsive to urate-lowering therapy, particularly when the serum urate is maintained at low levels below saturation concentrations [17, 18]. Understanding how improvements in clinical symptoms relate to changes in crystal deposition assessed by DECT following urate-lowering therapy will be of interest in future studies. Furthermore, the prognostic implications of MSU crystal deposition in people with well-controlled gout are currently unclear, and future prospective studies will be important to understand whether the presence of such deposition using advanced imaging tests predicts future flares or other clinical symptoms.

An important observation in this study was that not all individual remission domains were associated with DECT urate crystal deposition. Specifically, the pain domain was not associated with either presence or volume of DECT urate crystal deposition. A potential explanation for this observation is that pain due to gout is often maximal during gout flares [19], and many patients do not experience pain due to gout during intercritical periods [1, 20]. In this study population, more than two thirds of participants had a pain score of < 2 at the time of the study visit, using an instrument endorsed by OMERACT for assessment of pain in long-term gout studies [21]. Our findings regarding pain scores are similar to those described in studies of rheumatoid arthritis, in which the patient experience of pain is a frequent reason for discrepancy between physician and patient assessments of remission [22] and patients with ultrasound remission do not report lower pain scores [23].

The relationship between gout flares and DECT urate crystal measurement was also relatively weak. Although an association was observed in univariate analysis, gout flares were not independently associated with DECT urate crystal volume in the multivariable regression analysis which included all individual domains. Gout flares may be triggered even in the presence of small deposits of MSU crystals and require both MSU crystals plus an additional signal for NLRP3 inflammasome activation and initiation of the flare [24, 25]. Pain and flares are central concerns for patients with gout [26, 27], and inclusion of these domains within remission criteria is important to capture outcomes of relevance to patients.

While DECT is a well-validated and clinically useful imaging modality to assess urate crystal deposition in gout, the imaging protocol may have underestimated the total burden of MSU crystals in study participants. The study included scanning of all peripheral sites including the elbows and knees, which would capture the regions that are most frequently affected by MSU crystal deposition. The cut-off value of 3 mm diameter for reporting urate deposits was selected to avoid reporting of false positive deposits due to artifact [15]. However, it is possible that some small deposits may not have been captured in the analysis due to this cut-off value or the limits of DECT detection [28]. Similar studies using high-resolution ultrasound or the recently described multi-energy photon-counting CT might allow the use of small MSU deposit diameters/volumes with even higher accuracy [29, 30].

There are some limitations to this analysis. Due to the study design, there were some minor deviations from the published provisional remission criteria, specifically the number of times that serum urate, patient global assessment, and pain could be assessed. This may have over-estimated the number of participants fulfilling the provisional remission criteria. The purposeful sampling of the study (25% of patients with subcutaneous tophi and 50% with serum urate < 0.36 mmol/L) means that the proportion of people fulfilling the provisional remission criteria may not be generalizable to a community sample. Strengths include central reading of DECT by readers who were blinded to all clinical data, consistent clinical assessment and data collection, and prospective recruitment of participants.

## Conclusions

In people with gout established on allopurinol, a state of remission as defined by the provisional remission criteria is associated with less DECT urate crystal deposition. While this study provides some support for the validity of the provisional gout remission criteria, it has demonstrated that crystal deposition may still be present in some people achieving these criteria.

## Additional files

Additional file 1: **Table S1.** Overlaps between individual remission domains (*n* = 152 participants)*. **Table S2.** Combinations of individual remission domains (*n* = 152 participants)*. (DOCX 17 kb)

Additional file 2: **Figure S1.** Relationship between individual remission domains. The individual remission domains plots show the percentage of participants who fulfilled the relevant individual remission domain who fulfilled additional remission domains. (JPG 41 kb)

## Data Availability

The data that support the findings of this study are available from AstraZeneca, but restrictions apply to the availability of these data, which were used under license for the current study, and so are not publicly available. Data are however available from the authors upon reasonable request and with permission of AstraZeneca.

## Abbreviations

DECT: Dual-energy computed tomography
MSU: Monosodium urate
NLRP3: NACHT, LRR and PYD domains-containing protein 3
OMERACT: Outcome Measures in Rheumatology
