# Supplementary material for: Concurrent validity of provisional remission criteria for gout: a dual-energy CT study
Source: Arthritis Res Ther. 2019 Jun 21;21:150. doi: 10.1186/s13075-019-1941-8 (PMC6588898; doi:10.1186/s13075-019-1941-8)
Supplement: Supplementary file 1 — Table S1. Overlaps between individual remission domains (n = 152 participants)*. Table S2. Combinations of individual remission domains (n = 152 participants)*. (DOCX 17 kb) [file 13075_2019_1941_MOESM1_ESM.docx]

**Table S1. Overlaps between individual remission domains (n=152 participants).***

| **Individual remission domain** | **Serum urate (<0.36mmol/L)** | **Tophus (absence)** | **Flares (none in the last 12 months)** | **Pain due to gout (<2)** | **Patient global assessment of gout activity (<2)** |
| --- | --- | --- | --- | --- | --- |
| Serum urate (<0.36mmol/L) | - | 55 (36.2%) | 31 (20.4%) | 55 (36.1%) | 49 (32.2%) |
| Tophus (absence) | 55 (36.1%) | - | 54 (35.5%) | 73 (48.0%) | 63 (41.4%) |
| Flares (none in the last 12 months) | 31 (20.4%) | 54 (35.5%) | - | 60 (39.5%) | 54 (35.5%) |
| Pain due to gout (<2) | 55 (36.1%) | 73 (48.0%) | 60 (39.5%) | - | 77 (50.7%) |
| Patient global assessment of gout activity (<2) | 49 (32.2%) | 63 (41.4%) | 54 (35.5%) | 77 (50.7%) | - |

*****Data are presented as n (%) participants who fulfilled two individual remission domains.

**Table S2. Combinations of individual remission domains (n=152 participants).***

| **Serum urate** | **Patient global assessment** | **Tophus** | **Pain due to gout** | **Flares** | |
| --- | --- | --- | --- | --- | --- |
|  |  |  |  | **None** | **≥1** |
| **<0.36mmol/L** | <2 | Absent | <2 | 23 | 7 |
|  |  |  | ≥2 | 2 | 1 |
|  |  | Present | <2 | 12 | 3 |
|  |  |  | ≥2 | 0 | 1 |
|  | ≥2 | Absent | <2 | 2 | 2 |
|  |  |  | ≥2 | 4 | 8 |
|  |  | Present | <2 | 2 | 4 |
|  |  |  | ≥2 | 1 | 5 |
| **≥0.36mmol/L** | <2 | Absent | <2 | 14 | 13 |
|  |  |  | ≥2 | 1 | 2 |
|  |  | Present | <2 | 2 | 3 |
|  |  |  | ≥2 | 0 | 0 |
|  | ≥2 | Absent | <2 | 4 | 8 |
|  |  |  | ≥2 | 0 | 13 |
|  |  | Present | <2 | 1 | 4 |
|  |  |  | ≥2 | 2 | 8 |

*Data are presented as the number of participants.
