# Supplementary figures and images for: Concurrent validity of provisional remission criteria for gout: a dual-energy CT study
Source: Arthritis Res Ther. 2019 Jun 21;21:150. doi: 10.1186/s13075-019-1941-8 (PMC6588898; doi:10.1186/s13075-019-1941-8)

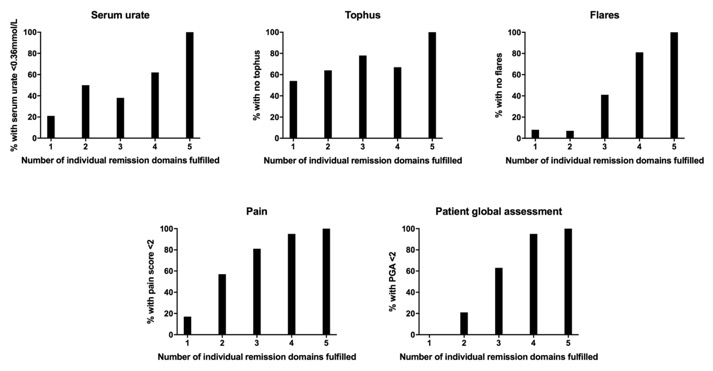

Supplement: Supplementary file 2 — Figure S1. Relationship between individual remission domains. The individual remission domains plots show the percentage of participants who fulfilled the relevant individual remission domain who fulfilled additional remission domains. (JPG 41 kb) [file 13075_2019_1941_MOESM2_ESM.jpg]
